# Malaria policy advisory committee to the WHO: conclusions and recommendations of fifth biannual meeting (March 2014)

**DOI:** 10.1186/1475-2875-13-253

**Published:** 2014-07-03

**Affiliations:** 1 Global Malaria Programme, World Health Organization, 20 Avenue Appia, CH-1211, Geneva 27, Switzerland

**Keywords:** WHO, Malaria, Policy-making, Mosquito control, Drug resistance, Surveillance, Elimination, Plasmodium falciparum, Plasmodium vivax

## Abstract

The Malaria Policy Advisory Committee to the World Health Organization (WHO) held its fifth meeting in Geneva, Switzerland from 12 to 14 March 2014. This article provides a summary of the discussions, conclusions and recommendations from that meeting.

Meeting sessions covered: maintaining universal coverage of long-lasting insecticidal nets; combining indoor residual spraying with long-lasting insecticidal nets; the sound management of old long-lasting insecticidal nets; malaria diagnosis in low transmission settings; the Global Technical Strategy for Malaria (2016 -2025); and Technical Expert Group updates on vector control, the RTS,S vaccine, the Malaria Treatment Guidelines, anti-malarial drug resistance and containment, and surveillance, monitoring and evaluation.

Policy statements, position statements, and guidelines that arise from the Malaria Policy Advisory Committee meeting conclusions and recommendations will be formally issued and disseminated to WHO Member States by the WHO Global Malaria Programme.

## Background

The Malaria Policy Advisory Committee (MPAC) to the World Health Organization (WHO) held its fifth meeting from 12 to 14 March 2014 in Geneva, Switzerland, following its meetings in February and September 2012, and March and September 2013 [1-4]. This article provides a summary of the discussions, conclusions and recommendations from that meeting as part of the *Malaria Journal* thematic series “WHO global malaria recommendations” [5].

The following sections of this article provide details and references for the background documents presented at the open sessions of the committee on: maintaining universal coverage of long-lasting insecticidal nets; combining indoor residual spraying with long-lasting insecticidal nets; the sound management of old long-lasting insecticidal nets; malaria diagnosis in low transmission settings; the Global Technical Strategy for Malaria (2016 -2025); and Technical Expert Group (TEG) updates on vector control, the RTS,S vaccine, the Malaria Treatment Guidelines, anti-malarial drug resistance and containment, and surveillance, monitoring and evaluation.

The MPAC discussion and recommendations related to these topics, which took place partially in closed session, are also included. MPAC decisions are reached by consensus [6]. The complete set of all MPAC meeting-related documents including background papers, presentations, and member declarations of interest can be found online on the MPAC website [7]. The next meeting of the MPAC will be 10 to 12 September 2014 [7].

### Report from the WHO global malaria programme

The acting Director of the WHO Global Malaria Programme (WHO-GMP) updated MPAC members on the major publications from WHO-GMP since its last meeting, including ‘Eliminating malaria: The long road to malaria elimination in Turkey’ [8], ‘Malaria control in humanitarian emergencies: an inter-agency field handbook’ [9], ‘Epidemiological approach for malaria control training manuals’ [10], and ‘WHO informal consultation on fever management in peripheral health care settings: A global review of evidence and practice’ [11].

The most significant of these recent publications is the World Malaria Report 2013 [12] which was launched in December in Washington DC. Among the highlights of the report are the following:

1. 59 out of 103 countries that had ongoing malaria transmission in 2000 are meeting the MDG target of reversing the incidence of malaria.

2. 52 of these are on track to meet Roll Back Malaria (RBM) and World Health Assembly targets of reducing malaria case incidence rates by 75% by 2015, including 8 countries in the WHO African Region.

3. In 41 countries, which account for 80% of all malaria cases, poor data quality or inconsistency in reporting methods makes it impossible to assess trends using reported data.

4. Between 2000 and 2012, estimated malaria mortality rates fell by 42% globally in all age groups and by 48% in children <5 years old.

5. Malaria mortality rates are projected to decrease by 52% in all ages, and by 60% in children under 5 years of age by 2015.

6. An estimated 3.3 million malaria deaths were averted between 2001 and 2012, and 69% of these lives saved were in the 10 countries with the highest malaria burden in 2000 - progress is being made where it matters.

7. About 3 million (90%) of the deaths averted between 2001 and 2012 are estimated to be in children under five years of age in sub-Saharan Africa. These account for 20% of the 15 million child deaths that are estimated to have been averted globally since 2000 through overall reductions in child mortality rates. Thus, decreases in malaria deaths have contributed substantially to progress towards achieving the target for MDG 4.

8. In 2012, financing of malaria programmes was estimated to be less than half of the estimated US$ 5.1 billion required globally.

9. Despite substantial progress in scaling-up programmes, 40% of households in sub-Saharan Africa did not have access to a single insecticide-treated net (ITN) in 2012 and millions still do not have access to diagnostic testing and artemisinin-based combination therapy (ACT).

10. As a result, an estimated 207 million cases (uncertainty interval, 135–287 million) and 627,000 malaria deaths (uncertainty interval, 473,000–789,000) are estimated to have occurred in 2012.

11. There is an urgent need to increase funding for malaria control and to expand programme coverage, in order to meet international targets for reducing malaria cases and deaths.

A full copy of the *World Malaria Report 2013* is available on the WHO-GMP website [12].

MPAC commended the work of WHO-GMP and its partners in the global malaria community in supporting countries in their efforts to monitor and reduce their malaria burden. The next report from WHO-GMP to MPAC in September 2014 will include updates from each of the WHO regions. It is anticipated that the new director of WHO-GMP will be recruited and announced, and ideally be in post, by that time.

### Maintaining universal coverage of long-lasting insecticidal nets

The Vector Control TEG (VC TEG) met for the second time from 24-26 February 2014 with three major outputs presented to MPAC. The first was draft recommendations for countries facing short-term gaps in long-lasting insecticidal net (LLIN) coverage. These draft recommendations were discussed at length but were ultimately not endorsed. While MPAC members acknowledged that projected funding shortfalls in some countries indicate the potential for temporary gaps in LLIN coverage, they were concerned that recommending a strategy of prioritized LLIN allocation might be misinterpreted as a move away from the goal of LLIN universal coverage. The proposed strategy could confuse Member States and detract from the principal task for ministries of health and their donors and partners, which is to secure sufficient resources to ensure universal coverage. These concerns were echoed during the meeting’s closed session. Instead MPAC recommended that WHO reiterate the goal of LLIN universal coverage in line with the outcomes from the September 2013 meeting [13]. In that previous recommendation, WHO outlined how universal coverage, defined as universal access to and use of LLINs, can be achieved and sustained operationally.

MPAC also suggested updating the universal coverage of LLINs recommendation in order to address confusion regarding LLIN procurement quantifications for mass campaigns. They suggested that calculation of the LLINs needed should be adjusted when quantifying at the population level, since many households have an odd number of members. Therefore, WHO should reiterate its recommendation that for procurement purposes (versus estimating population access, which is a different matter) countries should use an overall ratio of 1 LLIN for every 1.8 persons in the target population. Additional information, and the rationale behind the clarification, is provided in the note ‘Estimating population access to ITNs versus quantifying for procurement for mass campaigns’ which is now available on the WHO-GMP website [14].

### Combining indoor residual spraying with long-lasting insecticidal nets

Guidance for countries on combining indoor residual spraying (IRS) and LLINs was also presented by the VC TEG [15]. The reduction in disease burden of malaria in recent years has in a substantial part been attributed to the massive scale up of these two main vector control interventions, particularly in Africa south of the Sahara. A number of countries have deployed the two interventions in combination in an attempt to reduce transmission further; however, this action has not been informed by clear evidence.

Based on a review of published and unpublished cluster randomized trials conducted in Benin, Tanzania, The Gambia and Sudan, the VC TEG presented a summary of evidence on the combined use of IRS and LLINs which was reviewed and endorsed by MPAC following edits to improve order and clarity [16]. The main conclusions are summarized below:

1. In settings where there is high coverage with LLINs and they remain effective, IRS may have limited utility in reducing malaria morbidity and mortality. However, IRS may be implemented in areas where LLINs are being used as part of an insecticide resistance management strategy [17].

2. If LLINs and IRS are to be deployed together in the same geographical location for management of insecticide resistance, the IRS should use non-pyrethroid insecticides.

3. Malaria control and elimination programmes should prioritize delivering either LLINs or IRS at high coverage and to a high standard rather than introducing the second intervention as a means of compensating for deficiencies in the implementation of the first.

4. More evidence is needed on the effectiveness of combining IRS and LLIN in both high and low transmission settings. Evidence is also needed from different eco-epidemiological settings outside Africa.

5. All programmes in any transmission setting that invest in the combined use of LLINs and IRS should include a rigorous programme of monitoring and evaluation to determine whether the additional inputs have the desired impact. Countries that are already using both interventions should similarly undertake an evaluation of the effectiveness of the combination versus either LLINs or IRS alone.

A full copy of the VC TEG report, including the full review and summary of evidence, is available on the WHO-GMP website, in addition to the published WHO guidance note [16].

### Sound management of old long-lasting insecticidal nets

The final major VC TEG output presented to MPAC was ‘Recommendation on the sound management of old LLINs’ [18]. The VC TEG drew attention to this important issue because between 2004 and 2013, public health programmes distributed more than 700 million conventional ITNs and LLINs to communities. Currently, LLINs and the vast majority of their packaging (encompassing bags and baling materials) are made of non-biodegradable plastic material. However, most endemic countries do not currently have the resources for collection and waste disposal for these materials. The large-scale deployment of LLINs has given rise to questions on the most appropriate and cost-effective way to deal with the plastic waste that is accumulating in communities, particularly in countries where the malaria burden is high and where ministries of health seek to reach or sustain universal coverage of LLINs.

In summary, the VC TEG recommended that:

1. Residents should be advised through appropriate communication strategies to continue to use LLINs – even if they have holes – until another LLIN in better condition is available to replace it.

2. Residents should also be advised not to dispose of old LLINs (defined as those no longer used within households for the purpose of protecting individuals against malaria) in any water body, as the residual insecticide on the net can be toxic to aquatic organisms and especially to fish.

3. National malaria control and elimination programmes should only collect old LLINs if it has been ensured that: (a) communities are not left uncovered i.e. new LLINs are distributed to replace old ones, and (b) there is a suitable and sustainable plan in place for safe disposal of the collected material.

4. The collection of old LLINs should not divert the efforts and attention of malaria programmes away from their core duties, including the task of maintaining universal coverage.

5. If LLINs and their packaging are collected, the best option for their disposal is high-temperature incineration. They should not be burned in the open air. In the absence of such facilities, the recommended method of disposal is burial. Burial should be away from water sources and preferably in non-permeable soil, in line with ‘Recommendations on the sound management of packaging for long lasting insecticidal nets’ [19].

6. National malaria control and elimination programmes should work with national environment authorities to ensure that the information and recommendations in the recommendation are taken into consideration when formulating local guidance and regulations.

MPAC fully endorsed the VC TEG’s recommendations for the sound management of old LLINs, following discussion on how to improve the wording of the recommendations so that they would not unintentionally lead to a diversion of resources or responsibility. These changes are reflected in the summary above. The recommendations were approved pending edits by the WHO Secretariat to improve the clarity of the document prior to publication. The WHO recommendations for the sound management of old LLINs, including further details on the background and evidence base for the recommendations, are now available on the WHO-GMP website [20].

### Malaria diagnosis in low transmission settings

In recent years, the application of nucleic acid amplification (NAA)-based diagnostic tools to detect malaria for epidemiological surveys and research has increased significantly. Many different NAA assays are available with greater sensitivity than microscopy and rapid diagnostic tests (RDTs). NAA is the method of choice for confirming *Plasmodium knowlesi* and other zoonotic malaria infections.

In order to develop recommendations on the role of molecular diagnostic tests for malaria in low transmission areas, WHO/GMP convened an Evidence Review Group (ERG) on 16-18 December 2013 with the following objectives: (a) review current knowledge on the contribution of sub-microscopic parasitaemia to malaria transmission, particularly in areas with low transmission; (b) review the diagnostic performance, technical and resource requirements of available NAA methods for detecting low-density infections and to recommend the most suitable methods for population surveys and active case investigations; (c) review requirements to ensure quality for NAA methods and to build capacity to support their use in pre-elimination and elimination settings; (d) revise the current WHO recommendations for malaria diagnostic approaches in low transmission settings; and (e) discuss the malaria diagnostic research and development pipeline and reach consensus on preferred product characteristics for new diagnostic tools to meet public health needs for malaria elimination.

Based on the conclusions of the ERG [21], the MPAC endorsed the following recommendations [22]:

1. Quality-assured RDT and microscopy are the primary diagnostic tools for the confirmation and management of suspected cases of clinical malaria in all epidemiological situations, including areas of low transmission, due to their high diagnostic performance in detecting clinical malaria, their wide availability and relatively low cost. Similarly, RDT and microscopy are appropriate tools for routine malaria surveillance (of clinical cases) in the majority of malaria-endemic settings.

2. A number of NAA techniques are available and are more sensitive in detection of malaria compared to RDTs and microscopy. Generally, the use of more sensitive diagnostic tools should be considered only in low transmission settings where there is already widespread implementation of malaria diagnostic testing and treatment and low parasite prevalence rates (e.g. < 10%). Use of NAA-based methods should not divert resources away from malaria prevention and control interventions and strengthening of the health care services, including the surveillance system.

3. Submicroscopic *Plasmodium falciparum* and *Plasmodium vivax* infections are common in low as well as in high transmission settings. The use of NAA methods by malaria programs should be considered for epidemiological research and surveys aimed at mapping submicroscopic infections at low transmission intensity. There may also be a use for NAA methods for identifying foci for special intervention measures in elimination settings.

4. The majority of infections with asexual parasites have gametocytes detectable by molecular amplification methods, at low density not detectable by microscopy or RDTs. Most malaria infections (microscopic and submicroscopic) should be considered as potentially infectious and able to contribute to ongoing transmission. There is no need for routine detection of gametocytes using sensitive mRNA amplification methods in malaria surveys or clinical settings.

5. Common standards for nucleic acid based assays should be developed, including use of the WHO International *P. falciparum* DNA Standard for NAA assays and development of standards for other *Plasmodium* species, particularly *P. vivax* should be undertaken. A standard operating procedure should be developed which defines methods for sample collection, extraction, and the recommended equivalent quantity of blood to be added to the assay. Development of an international, external quality assurance system is strongly recommended to ensure that data obtained from NAAs are reliable and comparable.

6. In order to establish the role of serological assays in epidemiological assessments, there is a need for standardization and validation of reagents (antigens and controls), assay methodologies and analytical approaches.

Further details are available in the ERG meeting report, which is available in its entirety on the WHO-GMP website [23].

### Global technical strategy for malaria (2016 - 2025)

Following an expression of support by WHO Member States at the 2013 World Health Assembly in May last year, WHO-GMP is coordinating the development of the Global Technical Strategy for Malaria (GTS) for 2016-2025. Under the guidance of MPAC, the GTS will articulate the vision and goals for malaria over the next decade and bring together current policy recommendations in a comprehensive, evidence-based strategy for WHO Member States to use in developing their own strategies, wherever they are along the pathway to elimination.

Concurrent with the development of the GTS, the Roll Back Malaria (RBM) Partnership is coordinating the development of the Global Malaria Action Plan 2 (GMAP2) [24]. The GMAP2 will support the implementation of the GTS through global advocacy, resource mobilization, partner harmonization, the engagement of non-health sectors, as well as global, regional and country-level planning. Both documents are being developed in a synchronous, collaborative process, involving an overlap in steering committees, and will be jointly launched in 2015 to provide a strengthened platform for continuing malaria investments in the broader post-2015 development agenda.

The Chair of the GTS Steering Committee updated MPAC on the process to date [25], and provided the background on the proposed GTS targets [26], the overarching themes of the GTS and the pathway to elimination [27]. The GTS is being developed through an inclusive, country-driven approach. Based on a foundation of existing strategies, it will include input from consultations with WHO Regions, international experts, and country programmes. A series of regional consultations is scheduled between March and June 2014 and a public web consultation is scheduled for July.

MPAC members, and those observers present at the meeting, reviewed the first draft of the GTS and provided comments for the GTS Steering Committee and WHO-GMP Secretariat to consider prior to the start of the regional consultations. This discussion was further enriched via breakout groups and more detailed feedback on each of the GTS’s strategic directions.

WHO-GMP also provided MPAC with an update on progress of the technical brief on *P. vivax* malaria, which will consolidate all *P. vivax*-specific guidance into one document for the first time [28]. Based on the feedback from MPAC, the schedule for developing the *P. vivax* brief was modified so that it is aligned with the timeline for the GTS to facilitate the inclusion of relevant *P. vivax* guidance.

MPAC commended the GTS Steering Committee and WHO-GMP on progress to date, and the leadership of WHO-GMP and RBM on the close alignment of the processes for the GTS and GMAP2. MPAC members were especially supportive of the inclusive process that will involve country and regional input; these will be central to the development of both the GTS and the *P. vivax* brief, and critical to success. They acknowledged that as with any first draft of a document, greater development of the themes and targets was needed, but that it was useful to go into the regional consultations with a draft to provide a foundation for discussion.

The MPAC will next review the GTS electronically in August 2014, prior to its submission to the WHO Executive Board in September 2014.

### Updates from technical expert groups

In this session of the MPAC meeting, members received brief updates from each of MPAC’s standing expert groups.

#### Vector control advisory group

This joint expert group between the WHO Neglected Tropical Diseases Department and WHO-GMP functions to review and assess the public health value and “proof of principle” (epidemiological impact) of new tools, approaches and technologies; and to make recommendations on their use for vector control within the context of integrated vector management in multi-disease settings. The VCAG explained that one challenge to its programme of work has been the distinction that it assesses new classes of technology but is not involved in considering individual commercial products or the specifications of those products; this falls under the remit of WHOPES [29]. MPAC advised that the VCAG make its function and the process to submit a dossier for evaluation of innovative vector control tools more explicit on its webpage [30].

#### RTS,S/AS01 malaria vaccine

The Chair of the Joint Technical Expert Group (JTEG) on malaria vaccines entering pivotal phase 3 trials and beyond, established by the WHO Initiative for Vaccine Research (IVR) and WHO-GMP, provided an update to MPAC on its assessment and preparations for policy recommendations for the RTS,S/AS01 malaria vaccine [31]. Key analyses that are expected in 2014 include the results from the 30 months follow-up; the effect of a booster dose at 18 months; the effect of seasonality; and the breakdown of efficacy by age group within the 5-17 month age range. A detailed Q&A related to the RTS,S/AS01 vaccine is available on the JTEG webpage [32]. MPAC reiterated that RTS,S/AS01 will be evaluated as an addition to, not a replacement for, existing preventive and treatment measures, and that it is too early to define the potential public health role(s) of RTS,S/AS01. Depending on the results obtained in 2014, and on the regulatory submission timings, WHO will make the first malaria vaccine policy recommendations in late 2015.

#### WHO guidelines for the prevention and treatment of malaria

The Co-chair of the Chemotherapy TEG updated MPAC on progress with developing the third edition of the WHO Guidelines for the Prevention and Treatment of Malaria (MTGs) [33]. In brief, the process is on track; the updated systematic reviews of existing recommendations are now complete and the TEG met to review and reach consensus on the draft guidelines in November 2013. They will meet again in June 2014 to finalize the draft before beginning the process of internal and external review prior to presenting the MTGs to MPAC at its next meeting in September 2014. The Co-chair also presented the results of an online MTG end-user survey whereby the majority of respondents thought the MTGs were clearly written, appropriate in scope and size, and practically very useful. As a result, the TEG will retain the current MTG format for the new edition.

#### Drug resistance and containment

WHO-GMP updated MPAC on the latest drug resistance surveillance data and the agenda for the Drug Resistance and Containment (DRC) TEG meeting scheduled for 28-30 April 2014 in Geneva [34]. Although there have been admirable efforts to slow the spread of artemisinin resistance, efforts to contain resistance are proving to be more challenging than anticipated. There is now evidence for artemisinin resistant *P. falciparum* malaria in Cambodia, Vietnam, Lao PDR, Thailand, and Myanmar with independent emergence of new foci. MPAC, while acknowledging the hard work of the DRC TEG to date, urged it to focus much more strongly on strategies for artemisinin resistance containment at its April meeting. The TEG will report back to MPAC at its next meeting in September 2014.

#### Surveillance, monitoring and evaluation

WHO-GMP updated MPAC on progress with implementing the recommendations of the ERG on malaria burden estimation (ERG MBE) and in constituting the Surveillance, Monitoring and Evaluation (SME) TEG [35]. Although the call for nominations for members had been completed by the time of the MPAC meeting, not all proposed members had accepted their invitations and the member list was not announced. The membership is now posted on the WHO-GMP website [36] and their first meeting was convened from 14-16 May 2014 in Geneva. They will meet again in August 2014 and will report back to MPAC at its next meeting in September 2014.

## Discussion

The wording for recommendations were finalized by MPAC during their closed session following the two and a half days of open sessions; conclusions have been included in the summaries of the meeting sessions above, and links to the full set of meeting documents are provided as references. Position statements and policy recommendations made by the MPAC will be issued formally and disseminated to WHO Member States by WHO-GMP or the WHO Regional Offices. Conclusions and recommendations from MPAC meetings are published in the *Malaria Journal* as part of this series.

Feedback from the MPAC meeting will also be given to and received from the global malaria community at the RBM Board meeting in May 2014, through the publication of this article, and subsequent correspondence.

On-going engagement with and attendance by interested stakeholders at MPAC meetings continues to be encouraged. In addition to open registration for MPAC meetings, which will continue (via the WHO-GMP website starting in July 2014) and attendance by four standing observers (RBM, the Global Fund, UNICEF, Office of the UN Special Envoy for Financing the Health Millennium Development Goals and for Malaria), the active participation of seven rotating National Malaria Control Programme representatives and all six WHO Regional Malaria Advisors was strongly welcomed.

## Conclusion

The meeting feedback received from participants and observers [37], and MPAC members themselves, was very positive. Having met five times to date, the format of MPAC meetings and its feedback loops with other advisory bodies and stakeholders is fairly settled, although it remains an evolving process. WHO-GMP and the MPAC continue to welcome strongly any feedback, support, and suggestions for improvement to MPAC meetings from the global malaria community.

The next meeting of the MPAC will take place from 10 to 12 September 2014 in Geneva, Switzerland. Further information including the agenda and details on how to register will be made available in July 2014 on the MPAC page of the WHO-GMP website, although questions are welcome at any time [7].

## Abbreviations

ACT: Artemisinin-based combination therapy; ERG: Evidence Review Group; GMAP: Global Malaria Action Plan; GTS: Global Technical Strategy 2016-2025; IRS: Indoor Residual Spraying; ITN: Insecticide Treated Net; LLIN: Long-lasting insecticide treated nets; MPAC: Malaria Policy Advisory Committee; MTG: WHO Guidelines for the Prevention and Treatment of Malaria; NAA: Nucleic acid amplification; RBM: Roll Back Malaria; RDT: Rapid Diagnostic Test; TEG: Technical Expert Group; WHO-GMP: World Health Organization Global Malaria Programme.

## Competing interests

The authors declare that they have no competing interests.

## Authors’ contribution

All authors listed below have equally contributed to the article. All authors have read and approved the final version of the manuscript.

## Authors’ information

WHO Malaria Policy Advisory Committee Members

• Salim Abdulla, Ifakara Health Institute, Dar Es Salaam, United Republic of Tanzania

• Pedro Alonso, Centre for International Health and Research, Barcelona, Spain

• Fred Binka, University of Ghana, Accra, Ghana

• Patricia Graves, James Cook University, Cairns, Australia

• Brian Greenwood, London School of Hygiene and Tropical Medicine, London, UK

• Rose Leke, University of Yaoundé, Yaoundé, Cameroon

• Elfatih Malik, Ministry of Health, Gezira, Sudan

• Kevin Marsh, Kenya Medical Research Institute, Kilifi, Kenya

• Sylvia Meek, Malaria Consortium, London, UK

• Kamini Mendis, Colombo, Sri Lanka

• Allan Schapira, Legazpi City, Philippines

• Laurence Slutsker, Centers for Disease Control and Prevention, Atlanta, USA

• Marcel Tanner, Swiss Tropical and Public Health Institute, Basel, Switzerland

• Neena Valecha, National Institute of Malaria Research, New Delhi, India

• Nicholas White, Mahidol University, Bangkok, Thailand

WHO Malaria Policy Advisory Committee Secretariat

• Andrea Bosman, WHO Global Malaria Programme, Geneva, Switzerland

• Richard Cibulskis, WHO Global Malaria Programme, Geneva, Switzerland

• Bianca D’Souza, WHO Global Malaria Programme, Geneva, Switzerland and London School of Hygiene and Tropical Medicine, London, UK

• Abraham Mnzava, WHO Global Malaria Programme, Geneva, Switzerland

• John Reeder, WHO Global Malaria Programme, Geneva, Switzerland

• Pascal Ringwald, WHO Global Malaria Programme, Geneva, Switzerland

• Erin Shutes, WHO Global Malaria Programme, Geneva, Switzerland

• Chansuda Wongsrichanalai, WHO Global Malaria Programme, Geneva, Switzerland
